# Theranostic Digital Twins: An Indispensable Prerequisite for Personalized Cancer Care

**DOI:** 10.2967/jnumed.122.264929

**Published:** 2023-03

**Authors:** Julia Brosch-Lenz, Carlos Uribe, Arman Rahmim, Babak Saboury

**Affiliations:** BC Cancer Research Institute Vancouver, British Columbia, Canada

**TO THE EDITOR:** Recently, cancer patient digital twins have been astutely proposed as a conceptual framework with the vision of performing simulations for different treatment options before treatment is started (1). A digital twin is a virtual representation of the patient that uses mathematic and computational models to simulate personalized diagnosis and therapy. We strongly believe an optimal platform for more immediate actualization of the framework for cancer patient digital twins is theranostic nuclear medicine. Here, the theranostic digital twin (TDT) stands on the shoulders of two pathbreaking visions: precision medicine and complexity modeling.

Nuclear medicine imaging techniques and the inherent advantage of using theranostic pairs make treatment personalization based on physiologically based pharmacokinetic (PBPK) models readily feasible (2,3). Patient-specific optimization and personalization of radiopharmaceutical therapies (RPTs) can be obtained within the TDT framework, even before the first therapy cycle — a paradigm shift from existing one-size-fits-all therapies.

New technologic developments such as PET scanners with a long axial field of view provide a basis for extended-body PBPK modeling to strengthen TDTs (4). Artificial intelligence complements multiscale modeling (5,6) and offers the unique opportunity to improve every step of data acquisition, generation, and analysis. Theranostic imaging combined with artificial intelligence can be used to predict voxelwise absorbed doses and the effectiveness of RPTs (7).

The five pillars of cancer management can be divided into locoregional treatments (surgery, interventional radiology, and radiation therapy) and systemic treatments (oncology and RPT). Generally, the TDT not only serves as a prerequisite for personalized RPTs but will further support all five pillars. Residual disease after surgery or interventional radiology will result in an increased likelihood of local recurrence; theranostic imaging and TDTs can guide and verify the success. Although radiation therapy and brachytherapy are beneficial for localized diseases, systemic therapies present valuable options for metastasized diseases. TDTs can help in the personalization of systemic therapies by highlighting targets for medical oncology therapies using molecular imaging and PBPK modeling within the TDT. For RPTs, we believe that improvements in outcomes are possible when treatment with a TDT is optimized in terms of radioactivity, tracer amount, number of therapy cycles, and timing to an individual patient’s needs, conditions, and preconditions. There is evidence suggesting that with personalization, patient progression-free survival and overall survival can improve (8,9).

TDTs accept the challenge of combining existing multidisciplinary knowledge of cancer management, patient-specific data, and population-based models and create a 3-dimensional digital model representing the individual patient’s physiology and disease. Macroscopic observations (organ level, tissue level) are merged with microscopic effects (cell damage, tumor microenvironment), and the TDT evolves on the basis of (theranostic) imaging and physiologic information (i.e., PBPK). Incorporation of radiomics, personal physiology, and genomics enables rich TDT models. In the future, TDTs may be essential in determining whether a patient will benefit from one therapy and whether a different treatment is advisable, or TDTs may even predict how a particular treatment may inevitably fail, necessitating adjunctive and adjuvant therapies and interventions. TDTs will be updated continuously.

The TDT will facilitate and enhance therapeutic decisions to permit truly personalized cancer care. Being strongly committed to providing cancer patients with the best available treatment, we anticipate different solutions to enable TDTs to proliferate over the next decade. We encourage researchers to unlock the full potential of theranostics to support this important paradigm shift toward precision medicine.
